# Huichol Migrant Laborers and Pesticides: Structural Violence and Cultural Confounders

**DOI:** 10.1111/maq.12249

**Published:** 2016-01-27

**Authors:** Jennie Gamlin

**Affiliations:** ^1^ UCL Institute for Global Health University College London

**Keywords:** migrant labor, pesticides, Mexico, structural violence, Huichol

## Abstract

Every year, around two thousand Huichol families migrate from their homelands in the highlands of northwestern Mexico to the coastal region of Nayarit State, where they are employed on small plantations to pick and thread tobacco leaves. During their four‐month stay, they live, work, eat, and sleep in the open air next to the tobacco fields, exposing themselves to an unknown cocktail of pesticides all day, every day. In this article, I describe how these indigenous migrants are more at risk to pesticides because historical and contemporary structural factors ensure that they live and work in the way of harm. I discuss the economic, social, political, and racial inequalities that exist in their every‐day environment and how these forms of structural violence are mitigated by their intersection with local cultural contexts and their specific indigenous lifeworld.

Andrea, a Huichol migrant from the community of Cedros who has lived in the town of Santiago for the past four years, is feeding Itza, her six‐month‐old daughter. Itzá is chubby, covered in insect bites, mucus, and dirt. Lourdes, Andrea's sister is thin, with a bump that is small for her six months of pregnancy. “Where do you sleep?” I ask them. “Here's the hotel,” jokes Andrea, nodding toward the tarpaulin that hangs above a mountain of tobacco leaves beside them, and the two sisters, together with Lourdes's husband Lucio, continue discussing their living arrangements.

Lucio: Here nobody is comfortable, the kids get up and wander about, they pick things up and eat them. They pick up a stone and suck it, they have chemicals in them, dirty stuff they use here, gives you diarrhea, your mouth feels dry, I want to drink water but I don't as I worry it might cause me more harm, my head aches.

Lourdes: All the soil is poisoned. Last year, a smell reached me in the afternoons when I went out to pick. I felt dizzy. I vomited blood. A while ago I felt bad so I went to the clinic, they gave me a drip, they gave me medicine. These chemicals do us harm, give you a headache, you are out working and can give it to your baby, even make your baby ill. That's why it's born poorly, sleepy. Sometimes the kids get headaches when they are picking and carrying tobacco.

Itzá is sitting on the ground eating handfuls of dirt, the same soil Lucio has described as contaminated. “Here we are at risk,” he tells me:

There in the sierra everything is good, you pick up this and that and the only thing you get is cold. Here you have to buy water and this and that and you have to rent the land. There everything is nice and the land is good, but I can't go, the kids are studying and I still can't go.

I say goodbye telling Lourdes to take care of herself and she replies: “What can you do? We have to work!”

Many of the agrochemicals used on these plantations contain harmful organochloride or organophosphate compounds that have been shown to cause numerous forms of acute and chronic sickness and neurological harm, including cancers (Alavanja et al. 2004). They are known to have detrimental effects on human reproduction (since this is what they are designed to do to insects) (Petrelli and Figa‐Talamanca 2001; Thonneau et al. 1999), congenital malformations (Lacasana et al. 2006; Weselak et al. 2008), and potentially transgenerational effects (Nilsson et al. 2008).

For four months of each year, these two families live among tobacco plants that have been sprayed with up to 30 applications of agrochemicals. The particular nature of their working and living arrangements on these tobacco plantations means that these workers are at risk to pesticide exposures, and, as a family, they are particularly *in the way of harm*. I draw on the “violence continuum” (Bourgois 2002)—structural, everyday, and symbolic forms of violence—to discuss how social, political, and racial inequalities ensure that these migrants are particularly at risk from their continual exposure to pesticides and how their indigenous culture and lifeworld interact dynamically with these structures, confounding their effects on well‐being.^1^

The term “structural violence” is used to highlight how inequalities impact on well‐being and to identify “socially structured patterns of disease across population groups” (Quesada 2011). These afflictions may include mental and occupational health (Holmes 2007), infant mortality (Scheper‐Hughes 1992), substance abuse (Bourgois 2001), infectious diseases (Farmer 2001), or the many illnesses associated with poverty and marginalization. Structural violence is a political concept that locates the causes of affliction in global and historical inequalities and refers to the domination exerted systematically on everyone in particular social categories. Ethnography has demonstrated how this domination plays out through everyday and symbolic forms, which come together as a continuum of violence (Bourgois 2002). This continuum is explicitly linked to economic and political structures, historical events such as colonialism, and ongoing forms of domination, and they are violent because of the manner in which they cause physical, psychological, and emotional suffering.

Central to this concept is the idea that neoliberalism, viewed as a global form of governance, is immune and unaccountable to the suffering that it causes, making it a highly politicized approach to explaining affliction and one that can be hard to swallow within biomedicine, which tends to proffer an apolitical position (Green 2011). Rarely do we see a public health intervention that is directly critical of the neoliberal system or that seeks to address the structural inequaltities that generate ill health. While I concur with this explanation of the production of social inequality and its consequences, its use runs the risk of “depicting people as subjects of structural violence” (Biehl and Moran‐Thomas 2009:275). In so doing, it neglects both individual and collective agency, and the local and cultural specificities through which structural forms of violence are mediated. The geocultural context of Huichol tobacco migration sees the overlapping of exploitative forms of production linked to a global market economy with historical–traditional working relationships between mestizo farmers and indigenous workers who are bound by historical racial inequalities and the mediation of both forms of domination through indigenous cultural practices.

To the mestizo farmer, the visibility of cultural factors and apparent invisibility of structures of exploitation and discrimination sees local practices and beliefs confounding the role of structural inequalities, leaving culture to blame, or as Farmer (2001) suggests, culture becomes the “alibi” for structural violence. While the wixaritari^2^ are acutely aware of the poor conditions in which they work and live, the structures that perpetuate these conditions are not self‐evident, and what their employers see is an uncivilized and backward group of workers. The intersection of structural violence and culture also sees a dynamic process as Huichol workers respond to the various forms of violence that they experience.

I will argue that Huichol knowledge and practices relating to the risk of pesticides cannot be reduced to the metanarrative (Massé 2007) of structural violence but represent the interplay between this and their specific culture and circumstances. The local lived experiences that are exposed through ethnography link in with broad global economic and political powers. The challenge is to draw out the interlocking nature of these positions by identifying the roles of structure, culture, and agency.

Indigenous people worldwide are especially vulnerable to higher rates of maternal, infant, and child morbidity and mortality, heavy burdens of preventable infectious diseases, malnutrition, an increasing burden of social and life style–related diseases, the effects of environmental contamination, and a generally lower life expectancy (Gracey and King 2009). These health problems are caused by the particular nature of inequalities that affect them, the result of a combination of classical socioeconomic deficits and indigenous specific factors related to colonization, globalization, migration, loss of language, and culture (King et al. 2009). In Mexico, although colonialism officially ended in 1810, it has continued in its modern form for indigenous people who, in spite of their semi‐autonomous rights, are still governed by a mestizo state that is indifferent to their specific forms of social suffering.

On‐going cultural and social marginalization, poor‐quality education, and institutionalization of racial prejudice are modern‐day forms of state‐led structural violence that are the remnants of a colonial system (Freyermuth and Avendaño 2011; Smith‐Oka 2013). Present‐day political domination reproduces racial inequalities established by the Spanish colonial regime through education and welfare systems that stymie endogenous forms of development and distort existing community structures, while the non‐conformity and cultural practices of indigenous communities are still viewed as backward and uncivilized (Smith‐Oka 2013; Stephen 1999). The specific set of practices and beliefs that make up wixárika *costumbre*, the organizational paradigm of their community, are prominent markers of this difference.

Wixaritari live in accordance with a rich cosmological worldview, within which they share their lives and homes with spiritual beings, commune with gods, and organize their day‐to‐day activities, yearly events, and life‐cycle around the fulfillment of individual, family, and collective spiritual obligations. A fully trained *mara'akame* (shaman) is the spirit medium through whom people communicate with their deities, ask for good health, and learn of the obligations or rituals that are required to achieve this, ensuring adhesion to this specific organizational paradigm and fulfillment of religious obligations (Fajardo Santana 2007; Guttiérrez 2002).^3^

In spite of the prevailing culture of racial inequality, Huichols retain a moral high ground, bound to their reciprocal relationships with divinities and the natural environment that are reinforced through fiestas, ceremonies, rituals, offerings, and everyday living arrangements that continue to define life in their highland communities. As Liffman (2011:75) writes: “Instead of being dispossessed peasants, Huichols elevate themselves to the level of sacred governance by administering the means of both semiotic and ecological reproduction.” Although the largest proportion of ceremonial activity is confined to their highland communities and sacred sites, migrant workers bring their costumbre to the tobacco plantations. The visibility of their cultural and spiritual practices and beliefs serves to reinforce racist attitudes, as the local population is reminded of how their indigenous neighbors retain a pre‐modern, pagan life style, which, to their mestizo employers, is a symbol of primitiveness.

Migrant workers as a group are particularly vulnerable to the various forms of structural violence because of the temporary nature of their circumstances and because they work in spaces where their traditional forms of social support do not exist (Nguyen and Peschard 2003; Quesada et al. 2011). A growing body of research has demonstrated how different points of the violence continuum inflict pain and sickness on migrant farmworkers (Benson 2008; Duke 2011; Holmes 2007). These ethnographies coincide in illustrating how the combination of racial inequalities and economic exploitation are played out and internalized to the extent that they appear to be part of the natural order of things. It is this naturalization of social asymmetries that ensures their continuity, as indigenous people, migrant laborers, and other socially, racially, and economically marginalized groups become “complicit” in their subjugated position.

This symbolic violence, the symbolic demonstration of how structural factors undermine agency, as opposed to real/actual violence (Bourdieu 2001:34), is the self‐imposed belief that the marginalized somehow deserve their position in society. It is accompanied by attitudes of self‐blame such as shame, timidity, humiliation, and internalization and legitimation of hierarchy, which facilitate a continued unequal status quo. But this “systematic self‐depreciation,” an embodied form of domination, does not imply that the victim is to blame for his or her subordination. Rather, forms of domination are so embedded in the social structure and consciousness that they determine people's perception and actions (Bourdieu 2001:34). While this internalization is very real, for these Huichol workers, it is also accompanied by an acute sense of injustice.

These concepts have been used to describe ethnic divisions in farm labor camps in the United States (Benson 2008; Holmes 2007). Such ethnographic accounts of agricultural labor refer to the manner in which structural forms of violence inherent to the current global economy are played out as everyday forms of violence, such as racist interactions and exploitative employment, and how they become naturalized and effectively invisible, existing only in their embodied forms as physical, mental, and emotional suffering.

Here, I argue that the health problems and risks that Huichol tobacco migrants experience are compounded by the specific forms of violence that operate against them as an indigenous group and are sustained by their naturalized understanding of the risk, a knowledge seated within a context of everyday violence and mediated through a complex cosmological world‐view and its corresponding form of social organization.

## Methods

I first encountered Huichol tobacco migrants in 2006, when I visited Nayarit with a view to developing an epidemiological study of the health impacts of pesticides. While writing the proposal, I began to question whether this information would really be of use if the workers did not see pesticides as a health risk. I knew that wixaritari were not only highly marginalized but also that they maintained strong religious–cultural practices, suggesting that their world‐view and understanding of well‐being were likely to be very different than that of mestizos, and complex, so I began work on the development of a parallel ethnographic study that would explore these questions.

During the harvest seasons of 2010 and 2011 and on visits to communities in the highlands of Jalisco, I explored how migrant families from the traditional governorship of Cedros perceive of health and well‐being and what they know about the effects of pesticides, specifically seeking to understand what they knew about their effects on the reproductive process. I explored these questions mainly through semi‐structured interviews and participant observation. Interviews were conducted in Spanish or Huichol with the support of two bilingual research assistants.

The principal informants in this study are migrant laborers, many of whom spend their entire year working away from home, returning to the highlands only to plant maize and attend essential ceremonies. The men and women I interviewed are among the most marginalized of wixaritari families. Most have no other form of income; they are largely illiterate, usually from extremely distant communities that cannot be reached by road; and are frequently working to pay off debts.^4^ Migrant laboring is a way of life for many of them, and several women I spoke to had given birth while working in the fields. Many others had spent a large proportion of their lives moving with their young families between agricultural plantations, giving them ample opportunity to experience for themselves the effects of exposure to agrochemicals.

Between the first and second seasons I visited migrant families in their highland communities, talking to them in their homes and attending key events such as fiestas and ceremonies. I also conducted a number of interviews with tobacco farmers to ask about their use of pesticides and what they knew about harm. All first names used in this article and the names of towns (with the exception of Santiago) have been changed.

## Tobacco Production in *Tet+ata* 5: The Structural Determinants of Inequality

Tobacco farming on the coast of Nayarit works under the system of contract agriculture. Multinational tobacco companies, including Philip Morris and British American Tobacco, supply the seeds, dictate the production regime, and purchase the end product at a price to be determined by its final quality. The contract specifies the application of agrochemicals, establishes the price to be paid per *sarta* (length of threaded tobacco leaves) to the workers, and specifies conditions of the final exchange.

Essentially, the agreements are drawn up by and for the tobacco companies. For example, the insurance premium that is paid as part of the contract only covers the company's financial outlay, not losses to the farmer. If a crop is damaged due to bad weather—as happened in the spring of 2010—the company is not obliged to buy it from the farmer, but will be covered for its losses. These contracts mirror both the unequal terms on which small Mexican farmers must compete in an international marketplace, and the fact that the tobacco farmers’ union leader who is appointed to negotiate their contracts has long since been corrupted by the tobacco companies, a situation typical of the clientelistic relationships in Mexican politics (González de la Fuente 2007; Nieto 2011).

Fortunato, who has been farming tobacco in Nayarit for more than 40 years, explained to me how good tobacco is produced: soil preparation, sowing, the different fertilizers that must be used, and the pesticides that need to be applied. “A good leaf should be soft with these small brown spots on,” he tells me, and goes on to describe how the leaves are threaded and hung up to dry, making sure the air can circulate between the them.

“We hardly use agrochemicals,” he explains. There are a number of natural alternatives. Some farmers use bees to chase off the white fly or plant maize around the tobacco field to attract the insects; by taking care to ensure the plant does not get too wet, they can avoid the fatal blue mold. “Of course, though, if a plague comes in we have to use chemicals,” he adds.

Like his fellow tobacco farmers, Fortunato is reluctant to admit that he uses pesticides but talked at length about how poorly paid tobacco growing is. “So why *do* people still grow tobacco?” I ask. For these *ejidatarios* (farmers who cultivate *ejidos*, communally owned land), year‐on‐year earnings from crops such as beans, tomatoes, or maize are barely more than the cost of production and subsistence, leaving little to invest in seed the following season. Since tobacco companies pay production costs up‐front and shoulder a proportion of the financial risk, poor farmers can always afford to sow tobacco, even if the additional expenses incurred and low prices paid can leave them out of pocket.

In 2010, the set price per sarta was 9 pesos, but farmers paid their workers 10 or 11 pesos, in addition to the costs for pesticides used to deal with unexpected plagues such as white fly or the tobacco worm. The other reason why Nayarit farmers continue to grow tobacco—and a remnant of better times—is that social security contributions are paid, so when farmers eventually retire, they hope to receive a small pension from their tobacco work. The fall in commodity prices and accompanying deterioration of the value of labor sees some farmers intercalate tobacco production with tomatoes, maize, or beans. These are no more lucrative, and prices of vegetables together with government subsidies have continually deteriorated since the North American Free Trade Agreement was signed in the 1990s, making it harder for small Mexican farmers to compete with cheaper, mass‐produced, and genetically modified products. These structural inequalities have put a downward pressure not only on the profits of small farmers but also on the salaries they pay to workers. For both, their generalized poverty continues to tie them to season after season of hard work.

Once he has harvested and sold his own crop, Fortunato, like many Nayarit farmers, travels to Virginia to pick tobacco. His migrant income is his lifeline for six months of the year; there the sarta is paid at US$2.5 and the ton is bought at two, three, or four times the amount that is paid to Mexican producers.

In his ethnography of tobacco labor in North Carolina, Benson (2008:589) describes a situation not dissimilar to that lived in Santiago; fingers are caked in “gooey bits of tobacco,” which the Nayarit farmers refer to as *goma*, a diet of “beans, eggs, tortillas, and pickled carrots, onions, and chili peppers,” but most strikingly, “interlocking forms of subordination and marginalization.” Like these Santiago farmers, the author describes how North Carolina farmers take pride in being reasonable and responsible employers and commonly say that seasonal workers are part of the family, having employed the same people for several consecutive years. This working relationship is characterized by an apparent concern for the well‐being of workers, together with a deep‐seated belief in racial superiority.

The Nayarit farmers who I spoke to also describe the familiarity they have with their indigenous workers, an apparently benevolent attitude that serves to smooth over the racial superiority and socioeconomic exploitation that ultimately define their working relationships. Benson (2008:595–602) employs the term “faciality” to refer to the face of inferiority that is given to migrant workers and that becomes the justification for forms of everyday violence. Tobacco farmers on both sides of the border allow their employees to live and work in conditions that would be completely unacceptable for themselves or their families. Bartolo proudly explained to me how he had cared for the son of one of his Huichol employees while the boy studied secondary school in Santiago, yet this same employee, now nearly 70, sleeps in the shed where his patron stores dry tobacco and pesticide containers.

Like the U.S. tobacco farms that are profitable because they pay such low wages to Latino migrants, “tobacco production in Nayarit is only possible because indigenous people are willing to work for so little and in such poor conditions,” explains Saúl, a local activist who runs a support organization for small farmers. These days, there are more and more farmers with large extensions of land. These new *patrones* (employers) care less for the well‐being of their workers and more about their profit margin. During the course of my research, I learned of cases where workers had not been paid or had not been given water. Many of the older workers complained that the relationships with some ejidatarios was not like it used to be.

At the end of the season, leaves are smaller and fiddly to thread, consequentially it takes more time to produce one sarta. To ensure their employees finish the pick, some famers withhold payment until the entire field is cleaned, forcing workers to put in many hours for little pay during the hottest days of the year. This new culture of employer–employee relationships is becoming more common as a different and younger type of farmer with larger extensions of land and who cares little about their employees is replacing the traditional ejidatario: “Capital and its workforce are becoming more and more remote from each other” (Comaroff and Comaroff 2001).

Like the deteriorating production contracts that farmers must put up with and that see the continual devaluation of their produce, this deterioration of working conditions reflects structural changes that favor the profits of foreign companies. In the past, ejidos were this in their true sense, small towns operating around communally owned land. Since constitutional changes in 1992 legalized the sale and rent of ejidal lands, a new breed of farmers driven by profit began accumulating larger extensions of land and in the process, abandoning the traditional production relationships with workers.

## The Everyday Violence of Working and Living Conditions

The national and international structures that dictate the terms under which tobacco is produced in Nayarit ultimately impact on the living and working conditions of migrants, inflicting many of the everyday forms of violence that workers experience. In the 50 years since tobacco production took off in Nayarit, the living and working conditions of migrants have remained largely unchanged.

While other sectors such as tomato‐ and sugar cane–growing regions have seen the addition of low‐cost concrete shelters with running water and cooking facilities, Nayarit's tobacco migrants continue to live in extremely precarious conditions. Living, eating, and working on the tobacco fields all happen in exactly the same place, under the same palm shelter, and on the same small section of plantation. For a few years, one of the tobacco corporations provided canvas tents, some of which still remain, but mostly the migrant families hang a sheet or blanket, perhaps a mosquito net, and make this their home for the duration of their migration.

There are no sanitation facilities or running water in the fields, so the migrant families prepare their meals beside the tobacco plants, defecate in the open air, and eat their food with unwashed hands. With only a five‐gallon container of water for all the family's daily needs, even a basic level of hygiene is difficult to achieve. If they bathe, it is either in the contaminated irrigation canals that run alongside each plantation or once a week in the polluted River Santiago, so mostly they sleep covered in contaminated soil and sticky black tobacco resin. These conditions are markedly worse than those of other migrant laborers, most of whom benefit from shelter, cooking, and washing facilities away from the plantations.

This complete absence of housing, hygiene, and sanitation exposes migrant workers to an unknown cocktail of pesticide residue that is ingested, inhaled, and absorbed through the skin; fecal, airborne, and waterborne bacterial infections as well as eye and skin irritations relating to excessive exposure to the sun, insects, and dust—these living–working arrangements suit the piecework payment system and a household economy of poverty. A family of pickers usually begins work at first light, threading the last of the previous days’ tobacco while the morning dew dries. By 9 a.m., they will have completed their first pick and be taking a break for *almuerzo*, their first meal of the day. In the hottest part of the day they sit and thread the tobacco picked that morning, then work on the second pick sometime after the main meal of the day. Before moving from the tobacco leaves to their food, they will typically rinse their hands with half a cupful of water and wipe them dry on their clothing. After sunset, the laborers usually light a kerosene lamp and continue threading, often working well into the night before lying down near the piles of freshly picked leaves to sleep.

These migrants work such long hours because they are paid piecework as opposed to a standard daily rate, and they are able to do this because they live and work in exactly the same spot. Since there are no cooking, washing, or accommodation facilities to pay for, they are able to save as much of their income as possible. The conditions in which Nayarit tobacco workers are required to live and work is an everyday reminder of racial and structural inequality. A couple and their children typically work a 12–14‐hour day and earn between 250 and 400 pesos, far in excess of the daily rate of 70–100 pesos that a single person would earn as a day laborer. Farmers can pay them a low piecework rate knowing that by working long hours and picking as a family group with their children, they will earn a reasonable daily income. These conditions benefit the farmer and tobacco companies as their costs and those of the workers are negligible, enabling a degree of economic exploitation that would otherwise not be possible. Such everyday forms of violence deny migrants the opportunity for physical well‐being by exposing them to hazards that can cause sickness and death.

The conditions in which this almost entirely indigenous migrant workforce subsist are visibly more akin to the living spaces of farm animals than those of humans. They have no walls or a roof to protect them and are usually a considerable distance from a road or village, making workers dependent on their employers for daily supplies of water and tortillas or to gather them in the case of extreme weather. When Hurricane Kenna struck during the 2010 picking season, workers were brought in from the fields. Farmers boasted of valiantly driving their pick‐up trucks to the plantations in the middle of the stormy night to evacuate their workers. But instead of offering them dry spaces inside their homes, they housed them in sheds, on their terraces, in a flea‐infested semi‐constructed building, in the derelict town cinema that has lain in ruins for the past three decades, and in out‐buildings they use to store agrochemicals and tobacco, spaces that could only be considered fit for animals.

Indigenous workers are expected to endure living conditions that their employers would themselves find abhorrent because they are considered racially subordinate. This racism is embodied and internalized through a submissive attitude, a self‐valuation of inferiority, symbolic forms of violence. Subtle racism is an everyday form of violence that this population and the farmers who employ them have come to tolerate and naturalize, seeing it as the accepted order of things.

Many Huichols have given birth on the tobacco plantations, a situation farmers nonchalantly justify through, among other things, reference to a different set of cultural practices (“that's how they like it”), to physical resilience (“these indigenous women … they are very strong and resistant”), and a particular set of gender relations (“there are husbands who don't let their wives be seen by a doctor”). Thus, the view that that's how they like it is sustained by the specific faciality that farmers have given their employees, one that, like the racial stereotypes of Mexicans in southern United States, facilitates and justifies these everyday forms of violence.

The day after Hurricane Kenna, I was wandering around the ejido towns looking for families to enroll in my research. As I spoke to a group who were sheltered in a partly constructed building, the farmer came in and began to spray the rooms with insecticide. “Fleas,” he said, “this place is full of stray dogs.” In an interlinking of everyday and symbolic forms of violence, neither he nor his workers appeared to find anything unusual about the situation, there was no recognition of the appalling conditions in which these migrants would sleep, either by the farmer or his employees. As the farmer continued to spray, the migrants persevered with making beds for themselves by flattening piles of rubble and covering them with blankets.

## Experience of Illness and Normalization of Risk

Unsurprisingly, migrants who talked most about experiencing illness while working in contact with pesticides were families who spent more time working away. These are the young men, couples, and families for whom migrant laboring is a way of life. The tobacco harvest is particularly suited to family migration. The fact that accommodation and work are side by side facilitates childcare, while the *trabajo de ensarte* (threading tobacco leaves) that must be done sitting down, is easily managed by young children, nursing or pregnant women. Andrea, her sister Lourdes, and their families rarely returned to the sierra. Andrea spoke in detail about how pesticides had affected her and her children:

Andrea: Well, in the tobacco they all use poison, liquids, all that stuff, they say for the maggots, for the … what are they called? Plagues, those plague things. That's really difficult because it smells bad, it affects you, like your head aches and all that and sometimes blood comes out of your nose, that's it. …

JG: Do you know anyone who this has happened to?

Andrea: My youngest, it happens to her when she smells it, Estrella. When she smells that liquid, well, when they use liquid we have to go away so that we can't smell it, when the smell has gone we come back to the *ramada* (palm shelter). They also use it on tomatoes, when you are working you shouldn't eat tomatoes because they use liquid, I mean, sometimes that's why we get ill, something can die because of this and because they eat these fruits, I mean tomatoes, covered with liquids. … The boss tells us that it's not true, I just think they lie. …

Like Andrea, Bruno, who spends most of the year migrating, has seen the effects of pesticides on his children. He talked about how they had become ill while on the coast, illness that he blamed on their contact with contaminated soil. He tells me:

Of course, some kids have died after getting ill. The other day this boy [nods to his 18‐month‐old son] got ill soon after we arrived, it came from over there and he didn't like it, you know how they play in the soil, it lasted two days then he got a temperature and I was telling my wife to take him back [to the sierra] this week, if he gets ill it would be better to take him. But she didn't want to.

Many spoke of experiencing acute and immediate effects of exposure to pesticides, and there were several references to vomiting, headaches, and irritated eyes. Bruno also talked about goma, the black sticky resin that coats the leaves and rubs off onto worker's hands and clothing and how this irritated the eyes. Bruno's knowledge is embodied; he and his family have felt the effects of exposure to pesticides, yet their practices in relation to this appreciation of risk are determined by social and economic needs. His wife does not return to the sierra with their small sick son as she is needed to help gather and thread tobacco.

As Benson (2008) also notes in his study of tobacco migrants in southern United States, a series of interlocking factors ensure that these migrant workers are particularly in harm's way. The working and living conditions of Nayarit tobacco migrants guarantee that they are continually exposed to pesticides: The piecework system of payment and the fact that they have no accommodation away from the plantations makes it certain that they work all the daylight hours and into the night. There are no washing facilities with which to wash the goma off their hands and clothing, so they sleep covered in pesticide residue. Attempts to mitigate exposure, such as working fewer hours, renting a room, or using public washrooms on a regular basis would all come at an economic cost to the worker, either through expenditure or working time lost, potentially making their migration unviable. Again we see the interlocking forces of structural and everyday violence, as international corporations and legislation ultimately define unjust working conditions that include the impossibility of avoiding excessive exposure to pesticides.

## Understandings of Well‐being and Pesticides

Women who had worked while pregnant mostly spoke of unproblematic pregnancies and healthy children. Some expressed concern about the effects of work itself, but on the whole these positive experiences contradicted the idea that some aspects of work were harmful and normalized their life‐work experiences. Oscar and María had 10 children, two of whom died of infectious diseases in the sierra. María described to me how their trips to the coast were intermingled with pregnancies:

Oscar: When she was pregnant we went to work on the coast.

Maria: And I tell the women here, “How can you not go to work if I worked?” I didn't have any problem with my children, they were all born here, I went to work when I was pregnant and when I came back they were born. …

JG: And none were born on the coast?

Maria: No, I went pregnant and I came back pregnant and they were born here.

Apolonia spoke frankly, telling us that she simply did not know what effect pesticides might have. She described the good aspects of working on the plantations, telling us that “in truth I don't know if it's good or bad, nothing has ever happened to me and I have never been to work while pregnant, they work well and live well there.” In contrast, Andrea was more aware of the risks of exposure to agrochemicals. Four of her five children were born while she was working on plantations, the first in Zacatecas and the final three in Nayarit; in spite of this she did not identify this as a risk factor for harm during pregnancy:

JG: So the four youngest were born during picking season while you were working in agriculture?

Apolonia: Yes.

JG: And what do you think the risks are during pregnancy and for the unborn baby? I mean, a risk is something that could cause harm or problems. What do you know about things that could be risks for a pregnant woman and her baby?

Apolonia: Well, they shouldn't drink, that's it, if you drink it's a risk for pregnancy and also they shouldn't take pills and they should eat well, because if you don't eat well then they say a baby will be born with less weight, not big enough … they should eat well.

Apolonia's words probably reflect information she has received during ante‐natal visits, but few other informants received ante‐natal care. When I ask Olivia about the risks of pesticides she tells me: “Well, the baby could be born sick and with bad health. That's what they say but who knows if it's true.” Like Olivia, Cecilia, refers to having heard that pesticides could harm an unborn baby but expresses doubt about this.

Cecilia: Yes, like that [pregnant] I picked tobacco. It's that some men are really critical … if you don't work they get angry.

JG: And do you think this [pesticides] can affect an unborn baby?

Cecilia: Well yes, it's what they have told me, but I haven't seen anyone [ill]. But it is what they say, that you can get ill, but here [in the highlands] they are using them.

Pregnancy and birth are highly normalized events in the sierra. Like the rarámuri Indians, wixaritari women usually birth alone or are helped by only a family member; among both ethnic groups, maternal and infant mortality rates are among the highest in Mexico (Chopel 2014; Gamlin 2013). All but two of my female informants had experienced the death of one or more children. This violence of everyday life—exposure to pesticides while pregnant, giving birth alone, delivering babies alongside plantations, preventable infant and child morbidity and mortality—has been normalized. Such risks have become the natural and accepted order of things, so from everyday forms of violence we see the emergence of symbolic forms: the self‐depreciation and tolerance of intimidation that are the consequence of an acceptance of a structure of racial and gender inequality that has become embedded in their identities.

## Intersections of Structural Violence and Culture, Racism, and Dignity

These immediate and everyday forms of violence to which Huichol workers are exposed are violent because they cause mental, emotional, physical, and social suffering and illness. Through numerous essays that deal with this concept, Farmer (2005) indicates various “axes of oppression,” the layers on which structural violence operates to sustain inequality, injustices that become embodied in the experiences of people who live in poverty. These include structural forms of racism, gender inequality, legal, social, historical, political, and economic inequalities. The core of these axes is their ability to diminish the agency of their victims, effectively implying that they have become subjects of structural violence (Biehl and Moran‐Thomas 2009). There is certainly evidence of structural violence in its economic and political forms diminishing the agency of migrant workers, and the historical nature of this is omnipresent though everyday working relationships with their mestizo bosses, the epitome of ongoing unequal relationships with the mestizo society. However, these do not operate independently of culture nor do they annihilate agency; the relationship is far more complex. As Bhiel and Locke (2010:332) write: “People are not just the sum of these forces.”

Linking back to the lived experience of tobacco picking, although everyday forms of violence are present, the nature and circumstances of this work fit with the Huichol lifeworld and costumbre, in particular the fulfillment of religious obligations. This annual trip to Nayarit enables migrants to visit Hara'amaratise (sacred emergence site of wixaritari ancestors near the Nayarit coast). During their migration period, each family will make at least one visit to the coast to bathe in the sea and leave an offering, ask for fertility, life, or health. Furthermore, tobacco itself has a sacred association for the Huichol. Its darker strain, macuche tobacco, is grown in smaller quantities in the sierra to be used for ceremonial purposes, tied to babies’ wrists, and carried on pilgrimages (Gutiérrez 2002:121–122). This three‐month period of employment also fits neatly into the end of their ritual agricultural cycle, while bringing earnings that tide them over until the following season when their maize is again ready to eat. Growing and eating maize is central to Huichol culture, and this annual migration enables them to maintain their subsistence lifestyle, while at the same time fulfilling important religious obligations.

## Racism and Dignity

Huichols frequently scorn modern solutions to their lifestyle, preferring instead to live closer to the natural environment and retain traditional forms of subsistence, even if these imply much effort and at times hardship. For many years, they resisted the arrival of electricity; a proposed paved highway through the highlands was vehemently rejected and the new Crusade against Hunger program introduced by the government of Peña Nieto was initially refused by the community that participated in this study. In recent years, government programs have insisted on building toilets in their highland communities, resulting in three successive generations of empty, unused toilet structures: circular fiberglass shelters, square white iron boxes, and finally the Baño Digno, a concrete building complete with shower and “flushing” toilet, although none have running water. Women in the community of Cedros pointed out to me how the Baño Digno program serves only to suggest that open‐air washing and toilet practices lack dignity, while the Crusade against Hunger infers that they are “all dying of hunger.” These ideas are not true, they simply reflect the institutionalized belief that indigenous people and their lifestyles are backwards.

The conditions in which Huichol workers live while working on the coast—without running water and sanitation facilities—is a situation to which they are accustomed. But in their highland homes, they have access to clean natural water sources and pine and oak forests, they are surrounded by their deities, and they are not exposed to hazardous chemicals.

The sociocultural context and conditions of Santiago's ejidos, where Huichols are seen to occupy a lower rung of the social ladder than their mestizo neighbors, compounds their exposure to pesticides. The ingrained racism, wrought through centuries of political domination, is a specific form of structural violence that operates against them as an indigenous group and confers an unequal status extending beyond their relationship as employer and employee: Mestizo farmers assume working and living conditions that they themselves would find abhorrent to be acceptable for their indigenous workers. This classical contradiction within Mexican social history sees the valorization of ancient indigenous cultures and savagization of contemporary indigenous communities. Ancient cultures are a source of national pride, represented in prestigious museums and monuments, but present‐day indigenous practices and beliefs are considered backward and uncivilized and their religions are “pagan” (i.e., unenlightened), attitudes that reinforce mestizo belief in cultural superiority.

Many women have given birth on plantations, and when I asked farmers whether they saw this as a risk, their response was refer to the physical constitution of women, suggesting that “they are strong and resistant” and “they are brutes … imagine [cutting the umbilical cord], with a dirty knife!” In contrast, women described how health providers in Nayarit “don't treat them well” or “only attend people who are from Nayarit” [not indigenous people]. To avoid intimidating encounters, they fall back on their own trusted practices—in this case, birthing alone in the fields. Like the practice of open‐air sleeping and defecating, this is another example of the circular relationship between culture and structural violence, with cultural practices standing out for their visibility and operating as a cover for structural violence (Gamlin 2013).

Bourdieu and Wacquant (2004) explain how structural violence operates through the habitus to generate everyday and symbolic violence, with the latter being the embodiment of the structural form. Everyday violence exists in their ongoing conditions of exploitation, while symbolic violence is manifested in the manner in which these workers, in particular women, have rationalized and accepted the unequal nature of their lives. Although there is inevitably economic exploitation, and undoubtedly they are working in harm's way, the blow is softened by a set of cultural practices that accommodate the living and working conditions that they endure. The structural causes of the hazards—the unequal terms by which multinational tobacco companies contract employment and the underlying racisim inherent in the employment and living conditions to which they are exposed—have become invisible, with culture operating as a confounder of these structural injustices. As Massé (2007) suggests, “culture interferes with the structural, political and economic causes of suffering.”

Yet this dynamic interplay between structural violence and culture, racist attitudes and the naturalization of these also serve as a reinforcement of costumbre as Huichols defend themselves with a sense of ethnic and collective pride. In this case, through their costumbre, they retain a dignity and moral high ground that is evident in their “scorn [of] national power and market commodities, pale shadows of wixárika power and cultural goods” (Liffman 2011:76). These attitudes are culturally strengthening. As Comaroff (1991) writes: “Ethnicity is a set of relations, its content constructed in the course of historical process.” For wixaritari, this process has been marked by relations of racism and cultural oppression, forms of structural violence that appears to reinforce their practices and attitudes, a defense against the continual attacks on their dignity.

## Conclusion

In this article, I have discussed the structural forms of violence that ensure that these migrant laborers are working in harm's way. This exploitation is facilitated by a series of existing practices that form part of wixárika lifeworlds. The working conditions that they experience exist because of racial and economic inequalities but are endured because these migrants have normalized their status and accept social and racial inequality as the order of the mestizo world in which they must maneuver if they are to earn a living.

As medical and public health anthropologists and the wider body of professionals concerned with the well‐being and future of the world's indigenous people, we must reorient our understanding of risk and the focus of interventions toward the structural forces that put people in harm's way. It is the task of medical anthropologists to elucidate how these forms of structural violence embed themselves in existing sociocultural contexts and to demonstrate how specific lived experiences facilitate and normalize structural violence in a dynamic exchange between structure, culture, and agency.

## Notes

*Acknowledgments*. The research on which this article is based was jointly funded by the UK Economic and Social Research Council and Medical Research Council.

## Footnotes

1 I am using the term “confound” to mean “to mix‐up or mingle so that the elements become difficult to distinguish.”

2 Wixárika is the Huichol name for themselves; wixaritari is the plural (Huichols).

3 This cosmological world‐view and its accompaying rituals and practices are well documented by numerous authors, including the recent works of Neurath, Schaefer, Furst, Benítez and in earlier writings of Zingg, Lumholtz, and Preuss.

4 Families with official employment, such as in a school or clinic, those who work in a shop, small business owners, or families with many animals would not usually supplement their income as migrant laborers.

5 Tet+ata is the name given to the coastal region of Nayarit.
